# A Tool for Brain-Wide Quantitative Analysis of Molecular Data upon Projection into a Planar View of Choice

**DOI:** 10.3389/fnana.2017.00001

**Published:** 2017-01-17

**Authors:** Samme Vreysen, Isabelle Scheyltjens, Marie-Eve Laramée, Lutgarde Arckens

**Affiliations:** Laboratory of Neuroplasticity and Neuroproteomics, KU LeuvenLeuven, Belgium

**Keywords:** 3D reconstruction, pseudo *t*-test, non-parametric statistics, visual cortex, extrastriate areas, reorganization

## Abstract

Several techniques, allowing the reconstruction and visualization of functional, anatomical or molecular information from tissue and organ slices, have been developed over the years. Yet none allow direct comparison without reprocessing the same slices. Alternative methods using publicly available reference maps like the Allen Brain Atlas lack flexibility with respect to age and species. We propose a new approach to reconstruct a segmented region of interest from serial slices by projecting the optical density values representing a given molecular signal to a plane of view of choice, and to generalize the results into a reference map, which is built from the individual maps of all animals under study. Furthermore, to allow quantitative comparison between experimental conditions, a non-parametric pseudo *t*-test has been implemented. This new mapping tool was applied, optimized and validated making use of an *in situ* hybridization dataset that represents the spatiotemporal expression changes for the neuronal activity reporter gene *zif268*, in relation to cortical plasticity induced by monocular enucleation, covering the entire mouse visual cortex. The created top view maps of the mouse brain allow precisely delineating and interpreting 11 extrastriate areas surrounding mouse V1. As such, and because of the opportunity to create a planar projection of choice, these molecular maps can in the future easily be compared with functional or physiological imaging maps created with other techniques such as Ca^2+^, flavoprotein and optical imaging.

## Introduction

To fully understand brain processes, it is necessary to have a clear view on brain states at a specific time point and to be able to combine and comprehend the link between functional, anatomical and molecular information. Several techniques that allow visualization of one or more of these parameters have been developed over the years, but none of them allow for a direct comparison without reprocessing the same tissue. For example, the ever-growing interest of analyzing the response of the brain to specific stimulation and deprivation paradigms using functional imaging strategies, such as Ca^2+^, flavoprotein and optical imaging, is calling for methods that allow complementary gene and protein expression changes to be presented in a compatible format for comparative analysis. Furthermore, transparent brain techniques like iDISCO (Renier et al., 2014) and Clarity (Chung et al., 2013) have been developed to image the entire brain at once and, although they allow for whole brain anatomical investigations, they do not provide functional or physiological information.

Some techniques took advantage from public databases, created by organizations like the Allen Brain Atlas (ABA; http://www.brain-map.org), to map a 3D image generated from data extracted with *in situ* hybridization (Hibbard and Hawkins, 1984; Ford-Holevinski et al., 1991; Hecksher-Sørensen and Sharpe, 2001) and immunofluorescent protocols (Parfitt et al., 2012; Magee et al., 2015) or after noninvasive optical sectioning with MRI or CT (Höhne and Hanson, 1992; Klein et al., 2010), to match the 3D images of gene expression patterns within the public database. These databases, however, are generally limited to a specific set of genes, animals of a specific age (ABA: embryonic stage, P56) and to a limited number of species. Also, these reconstructions of molecular and structural data make it possible to compare with rodent fMRI data, but not with other functional imaging techniques, such as Ca^2+^, flavoprotein or optical imaging, because of the most common representation of such data in a top view fashion.

To overcome these limitations, we propose a new approach to build reference maps based on the animals analyzed in a given study, which makes it independent of age and species. Any type of data that can be transformed into optical density gradients can be used as input. This new tool then projects the data to a plane of view that can be comparable with functional brain images created with e.g., optical imaging. As in Hirokawa et al. (2008), the region of interest is segmented to create a grid, but in contrast to the standardized cortical box, the 3D location of the segment is preserved and projected to a plane of interest, which in brain research is often the horizontal plane. This results in a more realistic map where the shape and size of brain regions are preserved.

This technique was applied, optimized and validated on an *in situ* hybridization dataset consisting of slices at the level of the visual cortex from adult monocular enucleated (ME) mice. It has been previously described, based on serial 2D information about the expression of *zif268* mRNA, that there is an immediate dramatic reduction in the *zif268* mRNA expression levels in the monocular deprived visual areas, but that the visual cortex gradually recovers near normal activity levels over a time course of 7 weeks (Van Brussel et al., 2011; Nys et al., 2014). To illustrate the universal applicability of the tool, the technique was also applied to an immunofluorescent signal in brain sections to visualize transduction efficiency within the visual cortex with viral vector technology. Together these datasets illustrate how the new tool allows projecting and quantifying optical density-based patterns over a brain region of interest and to render views in a plane allowing comparison of the molecular findings to all kinds of brain imaging datasets.

## Materials and methods

We created a workflow written in Matlab (Matlab, 2015a, The MathWorks Inc., Natick, MA, USA) to register images of serial tissue sections to create planar representations of the tissue, and relevant signals inside, and to statistically compare the resulting images between different conditions. The tool is available as a Github repository under MIT License allowing users to use, copy, modify, merge, publish, distribute, sublicense, and/or sell copies of the tool without restrictions upon referring to this paper (https://github.com/sammevreysen/topview).

As a validation of the generalized method proposed here, we first applied the workflow on data from coronal brain sections processed for *in situ* hybridization for the immediate early activity reporter gene *zif268* in adult (P120) monocular enucleated (ME) C57Bl/6J mice of different post enucleation survival times (general vs. specific example parameters summarized in Table 1). This allowed us to cross-compare our findings with previous publications (Van Brussel et al., 2011; Nys et al., 2014). We next verified its applicability to an immunostaining dataset. Of note, series of coronal brain slices spreading over several millimeters along the antero-posterior axis were used in these examples, but any tissue of interest cut in any other anatomical plane could also be analyzed using this approach. Images acquired from *in situ* hybridization experiments, bright field or fluorescent microscopy, or any other image-based dataset could be used as long as the technique relies on optical density measurements for data analysis.

**Table 1:** **Overview of the specific parameters of the provided biological example data which are applied to the generalized method including derived measurements resulting from this specific set of chosen parameters**.

**Generalized method**	**Figures 1–4**	**Figures 5, 6**	**Figure 7**	**Figure 8**
Dataset	Dataset 1			Dataset 2
Cutting axis	Anterior-posterior			
Top edge 1	Pial surface			
Bottom edge 1	Border cortex layer VI and white matter	Border layer IV and layer V	Border cortex layer VI and white matter	
Top edge 2	n/a	Border layer VI and white matter	n/a	
Bottom edge 2	n/a	Border layer VI and white matter	n/a	
Histological/Anatomical landmarks	Anatomical border V2L, V1, V2ML and V2MM		Rhinal fissure and medial border V2MM	
Reference zone	Rectangular box in white matter			
Reference line	Midline of the coronal slice			
Number of equally spaced points	30		50	
Sampling grid resolution	0.100 mm A-P (serial sectioning)			
Distribution calculated perpendicular grid resolution	0.100 mm L-M (upper quartile) 0.143 mm L-M (upper fence)		0.096 mm L-M (upper quartile) 0.187 mm L-M (upper fence)	
Distribution calculated reduction histological and registration variation (Reference map, step 2)	0.057 mm L-M (median) 0.108 mm L-M (upper quartile) 0.235 mm L-M (upper fence)		0.040 mm L-M (median) 0.082 mm L-M (upper quartile) 0.181 mm L-M (upper fence)	
Bidirectional smoothing	0.2 mm			

### Image processing workflow

#### Slice registration

##### Manual specification of region of interest and histological landmarks

**Figure 1:**
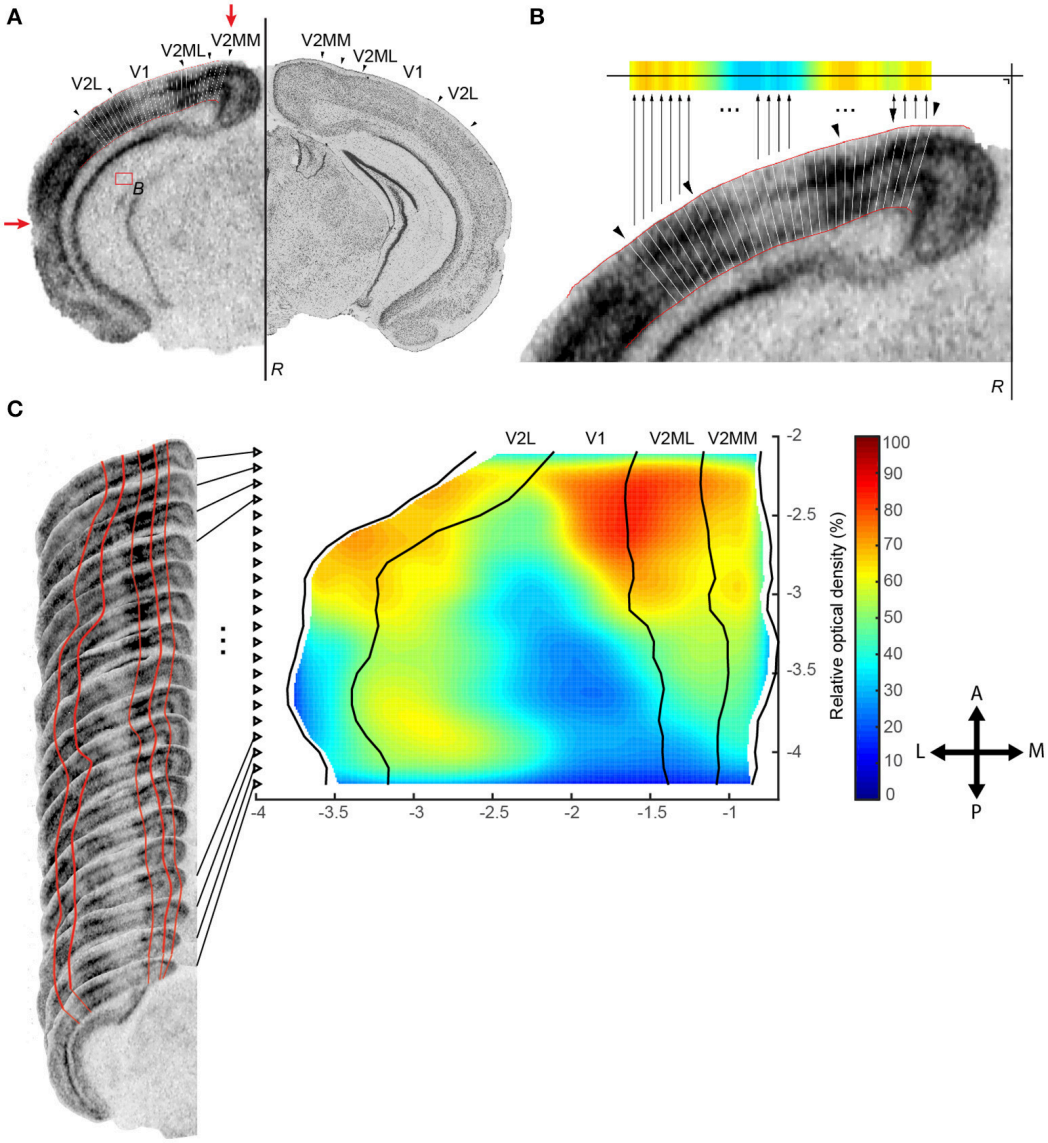
**Registration protocol. (A)** Black arrowheads indicate the interareal borders as defined upon Nissl counterstaining (right) and transferred to ISH data for *zif268* for the same slice (left). Areal (black arrows) and cortical layer borders (red lines) are manually registered together with a reference area *B* for normalization and a reference line *R* to create a planar projection. Red arrows indicate the registered positions of the outer borders used to create Figures 7, 8. **(B)** The mean relative optical density of each segment is calculated and projected onto the horizontal plane using reference line *R*. **(C)** The projection of each slice was aligned per animal to create an animal specific top view map. Axes unit in mm.

Several sections with a fixed interval are required to cover the span of a region of interest, here illustrated for a 100 μm series of coronal section of mouse brain tissue (Table 1). Each section first needs to be processed separately before all slices from one animal can be aligned along a specific axis based on their respective stereotaxic coordinates. For each slice metadata are manually created consisting of the condition, animal, filename, and corresponding stereotaxic coordinate. Next, the region of interest is registered by the user from the gray scale image by delineating the top and bottom edge of the structure (Figures 1A,B; red lines) and defining *L* histological landmarks *Z*^*l*^ (with *l* = {1, 2, …, *L*}) representing the edges of the different zones of interest within the structure (Figures 1A,B; black arrowheads). The top and bottom edge are smoothed by projecting the points onto a local regression line based on a weighted orthogonal least squares fit of the points (test_smooth_contours.m, v1.2, 20 Jul 2011, Tolga Birdal, Matlab Central #30793; wols.m, v1.2, 21 Mar 2011 Andrey Sokolov, Matlab Central #28894). A small rectangular reference region *B* is then manually assigned within the slice as representative of the background signal (Figure 1A), and the reference line *R* = {(*x, y*)|*ax*+*by*+*c* = 0} of the slice (Figure 1A) is manually traced to project the data in a later stage into a planar view.

##### Automatic calculation of optical density within segments

Next, to create column-like regions organized radially across the surface of the cortex, the algorithm divides the top and bottom edge of the region in *K* − 1 equal parts by interpolating *K* equally spaced points *P*^*k*^ (with *k* = {1, 2, …, *K*}) along their cumulative arc length and project these points back to their original curve (interparc.m, v1.3, 16 Aug 2012, John D'Errico, Matlab Central #34874). To ensure a good segmentation, the X-axis location of these K points should increase monotonically. In this way each quadrangular segment *S*^*k*^ is confined by the four points $\left[\begin{array}{cc} \begin{array}{cc} P_{top}^{k} & P_{top}^{k+1} \end{array} & \begin{array}{cc} P_{bottom}^{k+1} & P_{bottom}^{k} \end{array} \end{array}\right]$. The mean optical density (OD) is calculated from the pixels enclosed in this quadrangle *S*^*k*^ normalized by the reference region *B* using the formula $O{D_{S^{k}}}_{normalized}=1-\frac{\langle OD_{S^{k}}\rangle }{\langle OD_{B}\rangle }\;$ to remove high spatial frequency noise.

The distance from the reference line *R* of the slice to the center $C^{k}=\left(x_{k},y_{k}\right)$ between two adjacent points *P*^*k*^ and *P*^*k*+1^ representing a projection to a planar projection is computed using the formula (Equation 1) (Figure 1B):

(1) $$\left|\bar{C^{k}M}\right|=\frac{\left|ax_{k}+by_{k}+c\right|}{\sqrt{a^{2}+b^{2}}}$$

In summary, at this point we created a set of ordered pairs $\left\{O{D_{S^{k}}}_{normalized},\;C^{k}\right\}$ for each slice *n* (with *n* = {1, 2, …, *N*} slices per animal). We will refer to these quantities as *OD*^*n, k*^ and *C*^*n, k*^.

#### Reference map

**Figure 2:**
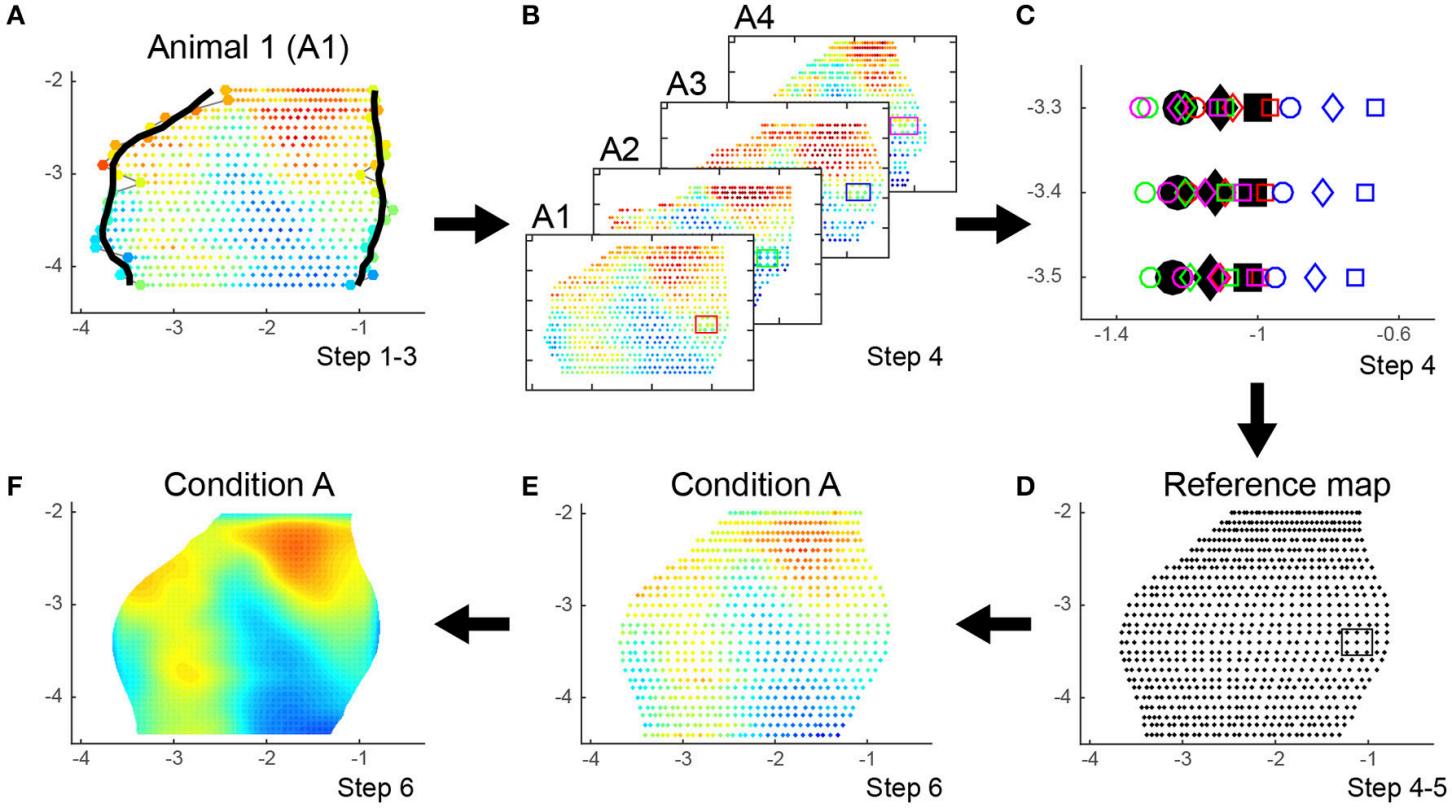
**Creation of an animal specific map, construction of reference map protocol and integration of individual animals in a condition specific map. (A)** Combine scattered points *C*^*n, k*^ from all K segments and all N slices per animal in one animal specific map and reduce both histological and registration variations by smoothing the medial and lateral edge of the map and recalculate each point *C*^*n, k*^ within each slice to fit the new outer boundaries by repositioning the reference line (step 1–2). **(B–D)** Create the reference map by combining the animal specific maps of all animals from all conditions (illustrated for animals 1–4 out of *N* = 26). Calculate the average position of *C*^*n, k*^ after smoothing across all animals to remove excessive variations between animals (step 3–4) and refine the reference map by smoothing along the axis of cutting (step 5). **(C)** displays the variation of nine corresponding points across four animals. The color of the markers matches the color of the rectangular boxes across the selected points in **(B)**. The shape of the marker defines its position along the lateral-medial axis (circle, diamond, square). Black markers show the average position of the nine points after smoothing across the four animals and along the axis of cutting. **(D)** Refined reference map. The points within the black rectangular box correspond to the black markers displayed in **(C)**. **(E)** Each animal specific map is non-parametrically warped to the reference map. Maps from animals belonging to the same condition are merged by calculating the mean intensity value across all animals (step 6). This condition specific map is interpolated to enhance visualization as shown in **(F)**.

To reconstruct the region of interest, animal specific maps as well as a reference map are built from all registered slices by applying the following algorithm. All steps are illustrated in Figures 1, 2 and Table 1:

1. Create a map for each animal based on the scattered points *C*^*n, k*^ with corresponding OD values from all N slices per animal (Figures 1C, 2A).
2. Smooth the position of medial and lateral edges of the map using an algorithm that penalizes points that deviate from the smooth curve created by surrounding points. Therefore, we implemented a moving average weighted by the complement of the relative second derivative of the symmetrically padded version of these edges. In detail (script available as Data Sheet 1):
  - A curve is created from the X-axis position of the *N* points (*X*_1_, *X*_2_…*X*_*N*_) outlining the medial or lateral edge.
  - This curve is symmetrically padded by mirroring the second (*X*_2_) and before last point (*X*_*N*−1_) in front (*X*_−1_) or at the back (*X*_*N*+1_) of the curve, respectively, creating a curve with *N* + 2 points.
  - From this extended curve the second derivative for each point *D*^*n*^ is calculated (with *n* = 1, 2 …*N*) using the formula $D^{n}=2X_{n}-X_{n-1}-X_{n+1}$ and made relative by dividing all values to the maximal value of the second derivative (*D*^*n*^ = *D*^*n*^/max(*D*^1, 2 …*N*^)).
  - The complement of these relative values ($D_{complement}^{n}=1-D^{n}$) are finally used as weights in a weighted moving average to penalize large deviations (“bumps”) in the curvature using formula (Equation 2) with window size *w*:
    (2) $${X^{\prime }}_{n}=\frac{\sum_{i=n-\left\lfloor w/2\right\rfloor }^{n\text{+}\left\lfloor w/2\right\rfloor }\;D_{complement}^{i}\;\cdot \;X_{i}}{\sum_{i\;=\;n-\left\lfloor w/2\right\rfloor }^{n\text{+}\left\lfloor w/2\right\rfloor }\;D_{complement}^{i}}\;\left(with\;1\leq i\leq N\right)$$
3. Based on the smoothed outline of the region of interest the position of each point *C*^*n, k*^ and *Z*^*n, l*^ within each slice is recalculated to fit the new outer boundaries by repositioning the reference line. If this is not possible because the morphology is too distorted, the new positions of *C*^*n, k*^ and *Z*^*n, l*^ are inferred from the surrounding slices by interpolation (inpaint_nans.m, v1.1, 13 Aug 2012, John D'Errico, Matlab Central #4551). This smoothing step reduces both histological and registration variations within the same animal (Figure 2A, Table 1).
4. Create the *reference map* by combining the maps of all animals from all conditions: calculate the average position after smoothing of *C*^*n, k*^ and *Z*^*n, l*^ across all animals using a moving average weighted by the complement of the symmetrically padded relative second derivative to remove excessive variations between animals (Figures 2B,C).
5. Refine the reference map by smoothing along the axis of cutting using the same weighted moving average algorithm from step 2 on *C*^•, *k*^ and *Z*^•, *l*^ (• means across all n slices), create a grid along that axis with a resolution equal to the sampling interval of the slices and linearly interpolate any missing data points on the grid (Figures 2C,D).
6. For each animal, assign each *OD*^*n, k*^ from this animal to its corresponding average position *C*^*n, k*^ in the reference map resulting in the map *A*_*n*_. Merge the maps from different animals belonging to the same condition *A* by calculating the mean intensity value across all animals for all scattered points *i* = (*x, y*), which creates the map $\bar{A\left(i\right)}$ (Figure 2E). This map will be interpolated to a resolution of 1/10 of the grid size to enhance visualization. Finally, the map is bidirectionally smoothed using a Gaussian kernel of full width at half-maximum (FWHM) with a window double the size of the sampling interval of the slices (Figure 2F).
7. Calculate the difference image Δ between conditions *A* and *B* for each pixel *i* in $\bar{A\left(i\right)}$ and $\bar{B\left(i\right)}$.

#### Randomization test with pseudo *t* statistics

To determine whether the differences between two conditions are statistically significant, we adapted and implemented the randomization test with pseudo *t* statistics created by Holmes et al. (1996). This algorithm is well known in fMRI and PET scan analyses to compare images originating from two independent conditions. A non-parametric testing approach is favorable above a parametric testing approach because many assumptions and approximations may not be true, such as a known probability distribution of the pixel values. Also a low number of subjects results in noisy statistic images with a low degree of freedom. The formal assumptions of a parametric testing approach are replaced by a computationally expensive approach using a randomization test. The principle behind this non-parametric testing approach is based upon a randomization and permutation test with pseudo *t* statistics (Holmes et al., 1996). We can assume that if condition *A* is truly not different from condition *B* this labeling is artificial, hence any other combination of assigning the animals into the two conditions would lead to an equally plausible statistic image. In this way, we create the null hypothesis $H_{0}^{i}$ stating that the labeling is arbitrary for every pixel *i* = (*x, y*), meaning that the observed statistical image originated from a random labeling of the animals to condition *A* or *B*.

We label a (different) number *N*_*A*_ and *N*_*B*_ of animals to be part of condition *A* or condition *B* respectively and build a statistic image based on those labels. For every pixel *i* = (*x, y*) we calculate the difference image Δ between the two condition specific maps *A* and *B* and variance image $S_{A}^{2}$ and $S_{B}^{2}$ from condition A and B respectively:

(3) $$\Delta \left(i\right)=\bar{A}\left(i\right)-\bar{B}(i)$$

(4) $$S_{A}^{2}\left(i\right)=\frac{1}{N_{A}-1}{\sum_{n=1}^{N_{A}}\left(A_{n}\left(i\right)-\bar{A}\left(i\right)\right)}^{2}$$

Expecting the true error variance to be smooth, we apply a convolution to the variance images using a Gaussian kernel of full width at half-maximum (FWHM), resulting in the smoothed variance images ${S^{\prime }}_{A}^{2}$ and ${S^{\prime }}_{B}^{2}$. We then derive the pseudo *t* statistic image *T* without assuming equal variances:

(5) $$T\left(i\right)=\frac{\Delta \left(i\right)}{\sqrt{\frac{{S^{\prime }}_{A}^{2}}{N_{A}}+\frac{{S^{\prime }}_{B}^{2}}{N_{B}}}}$$

We implemented the step-down test to calculate the adjusted *p*-values images, in short: we sort the pixels of the observed statistic image from small to large and create a successive minima and maxima image for each permutation based on the sorted list of the observed statistic values. Next, we count per pixel the number of permutations having a successive minimum (maximum) value smaller (bigger) than the observed value of that pixel. *p*-values are computed by dividing these counts by the total number of permutations *N* = _(*N_A_*+*N_B_*)_*C_N_A__* = *N_A_*!/[(*N_A_* + *N_B_*)!]^2^ and then the adjusted *p*-value image is calculated by enforcing monotonicity along the sorted list of observed statistic values. Both adjusted *p* -value images for activation and deactivation will have *p* -values between 1/*N* and 1. Assuming a significance level α = 0.05 we can now threshold these images at *p* = α for one tailed (de)activation and *p* = α/2 for two tailed comparison. Subsequently, the minimum number of animals for each condition is *N*_*A*_ = *N*_*B*_ = 3 resulting in a minimal possible *p* -value of 1/*N* = 1/20 = 0.05 (Table 2). As negative control we show the comparison of two groups of animals (A and B, column 1 and 2) belonging to the same condition (Supplementary Figure 1A). To validate the false alarm rate set at α = 0.05 we created 1000 times normal distributed random data for 6 animals split over 2 conditions (mean OD value = 50%, standard deviation = 20%, grid = 30 × 25). We calculated the family-wise error rate (FWER) as the percentage of these 1000 repetitions in which we could detect at least one significant pixel with *p*_*activation*_ ≤ α/2 = 0.025 and *p*_*deactivation*_ ≤ α/2 = 0.025. The FWER was (20 + 28)/1000 = 0.048 which matches closely the chosen alpha rate of 0.05 (Supplementary Figure 1B). This finding closely matches the observation described by Nichols and Holmes (2003) that this procedure maintains a strong control over the FWER.

**Table 2:** **Number of permutations and corresponding lowest possible ***p*** -value depending on the group size assuming the groups to be equal in size**.

**Number of animals per group (equal group size)**	**Number of permutations**	**Lowest *p* -value**
3	20	0.0500
4	70	0.0143
5	252	0.0040
6	924	0.0011
7	3432	0.0003
8	12870	7.77e-05
9	48620	2.06e-05
10	184756	5.41e-06

*Increasing the number of animals exponentially increases the computational burden*.

### Example data set 1: *in situ* hybridization

**Figure 3:**
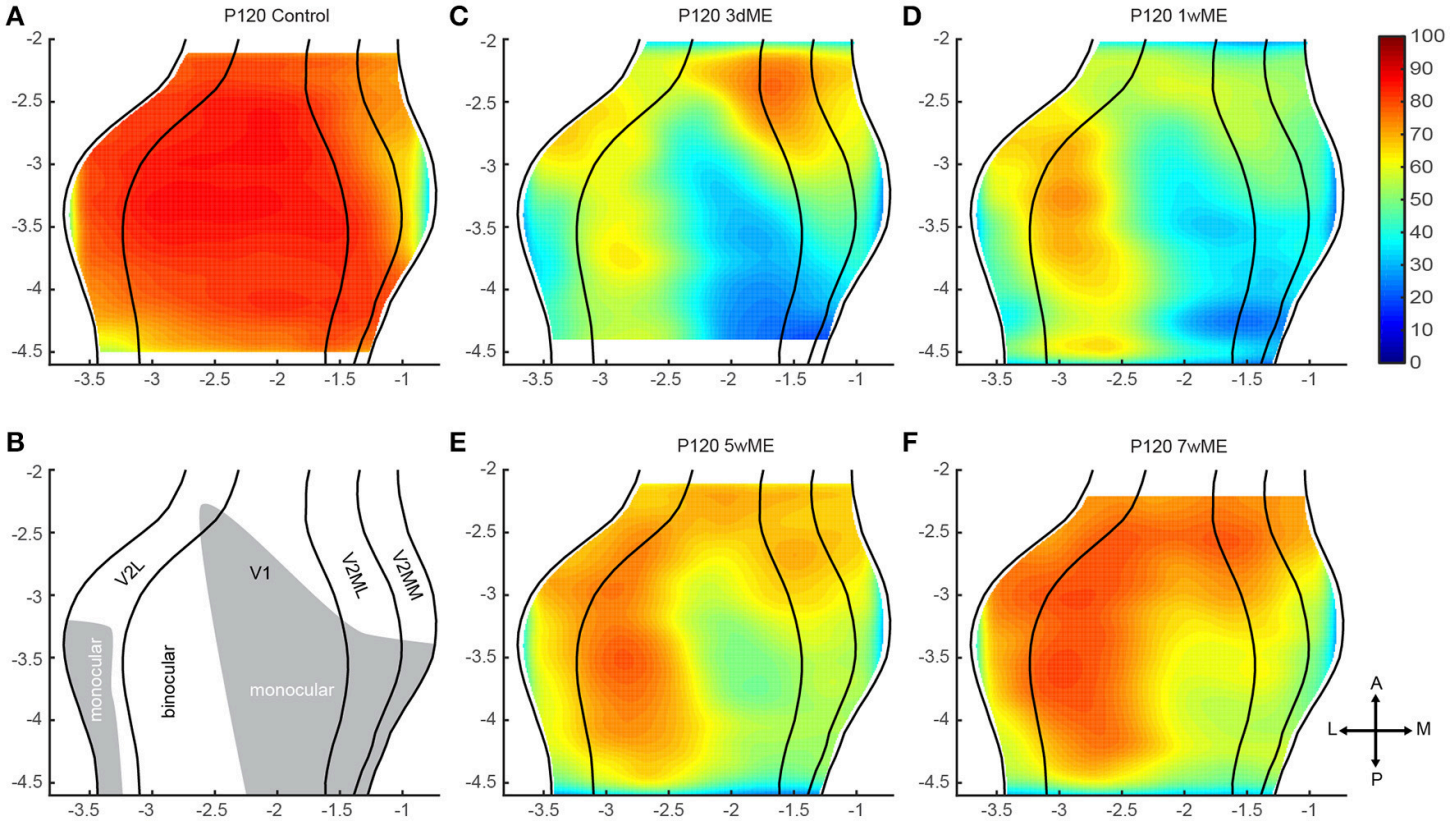
**Top view representation of the relative ***zif268*** expression of the visual cortex of the mouse in (A)** adult control animals and after **(C–F)** 3 days, 1, 5, and 7 weeks of monocular enucleation (ME). **(B)** Illustration of the delineated visual areal borders for V2L, V1, V2ML, and V2MM (lateral to medial, black lines) based upon the cytoarchitecture (Nissl). Based upon the drop of *zif268* expression visible in the map of 1wME mice the monocular (gray area) and binocular zones (white area) within the visual cortex were distinguished. **(C–F)** A sharp decrease of expression is visible after 3 days of ME with a gradual and partial recovery after 7 weeks of ME. Axes unit in mm.

**Figure 4:**
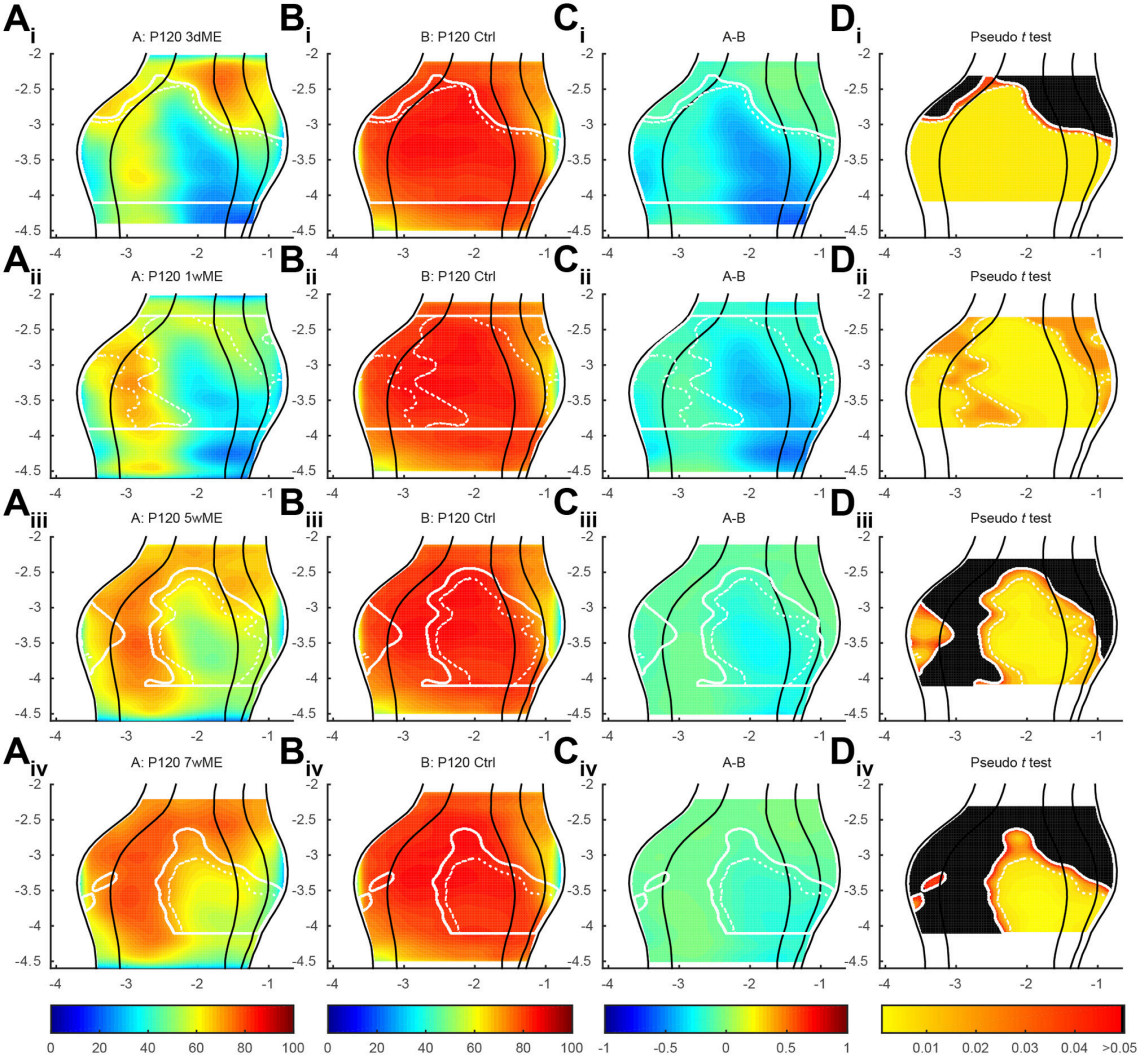
**(A_**i-iv**_)** Top view representation of the relative *zif268* expression of the visual cortex of the mouse for the conditions P120 3d, 1w, 5w, and 7wME (condition A) vs. **(B_i-iv_)** P120 control (condition B) together with their **(C_i-iv_)** relative difference between conditions A and B and **(D_i-iv_)** one-tailed deactivation pseudo *t* -test image. Overlay white solid and dashed contour lines show the boundary where the null hypothesis has been rejected with α = 0.05 and α = 0.01, respectively. ME results in the most pronounced decrease in *zif268* expression in the posterior aspect of the visual cortex which recovers over time except for the monocular zone. At 1wME there is a delayed suppression in the most anterior region of the visual cortex, which recovers swiftly. In the extrastriate areas, the initial reduced patch in V2L reactivates almost completely in contrast to the posterior halves of V2ML and V2MM, which do not reach normal values again. In all visual areas the binocular zone is less reduced compared to the monocular zone and recovers fully after 5 weeks through a medial expansion toward the monocular zone of V1 (medial shift of solid white line at monocular-binocular border of V1). Axes unit in mm and orientation as used in previous figures.

**Figure 5:**
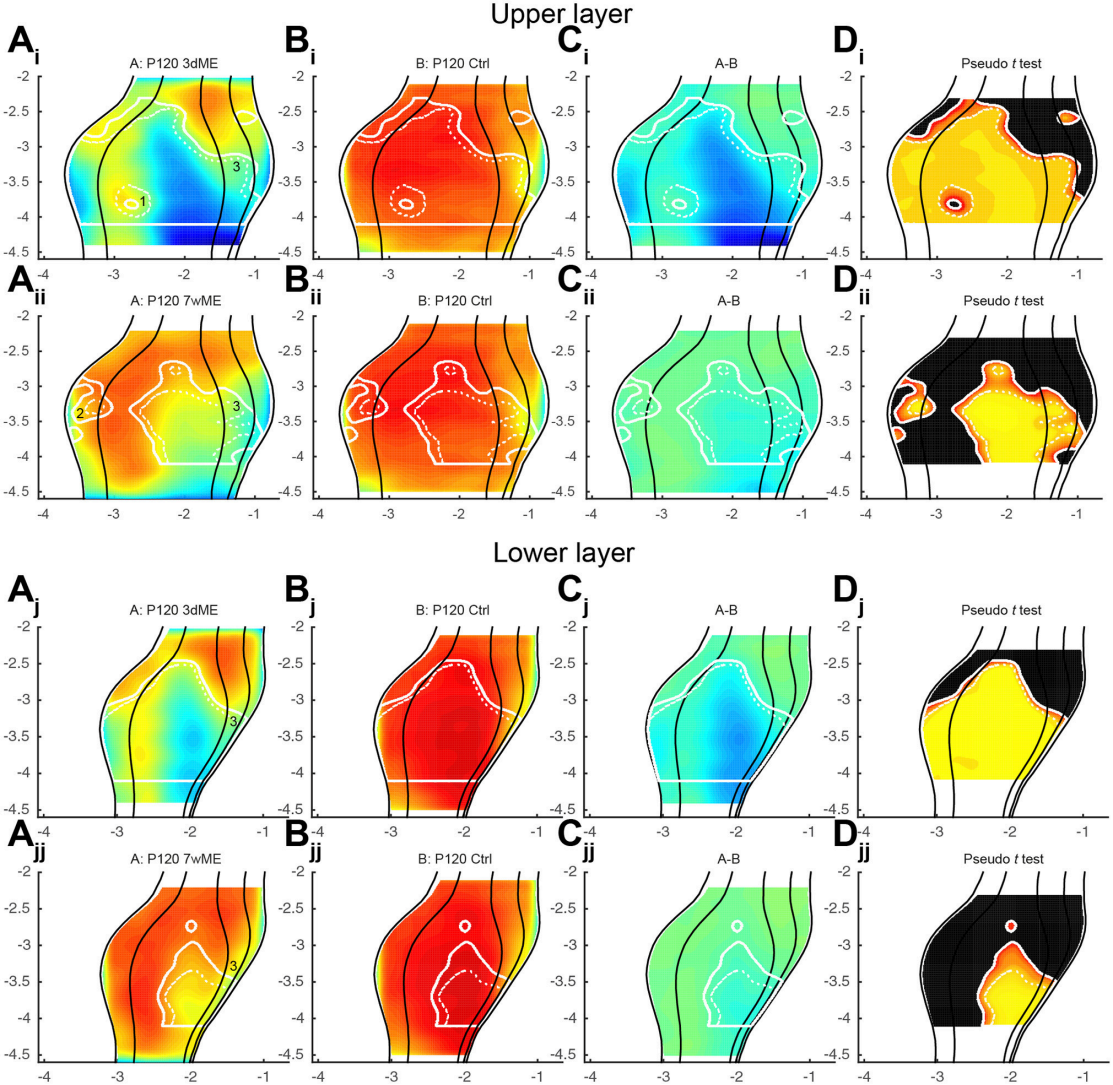
**Expression maps of the visual cortex for *zif268* split up for (A_i-ii_–D_i-ii_)** upper (layer I–IV) and **(A_j-jj_–D_j, jj_)** lower (layer V–VI) layers comparing 3dME and 7wME with the control condition. Both upper and lower layers show similar *zif268* expression levels, except for three patches: (1; **A_*i*_**) in the upper layer in the posterior binocular zone of V1 after 3dME, (2; **A_ii_**) in the upper layer in V2L after 7wME and (3; **A_i, ii, j, jj_**) after 7wME the upper and lower layers have a dissimilar patch in the medial extrastriate cortex. Panels and axes legends are the same as in Figure 4.

**Figure 6:**
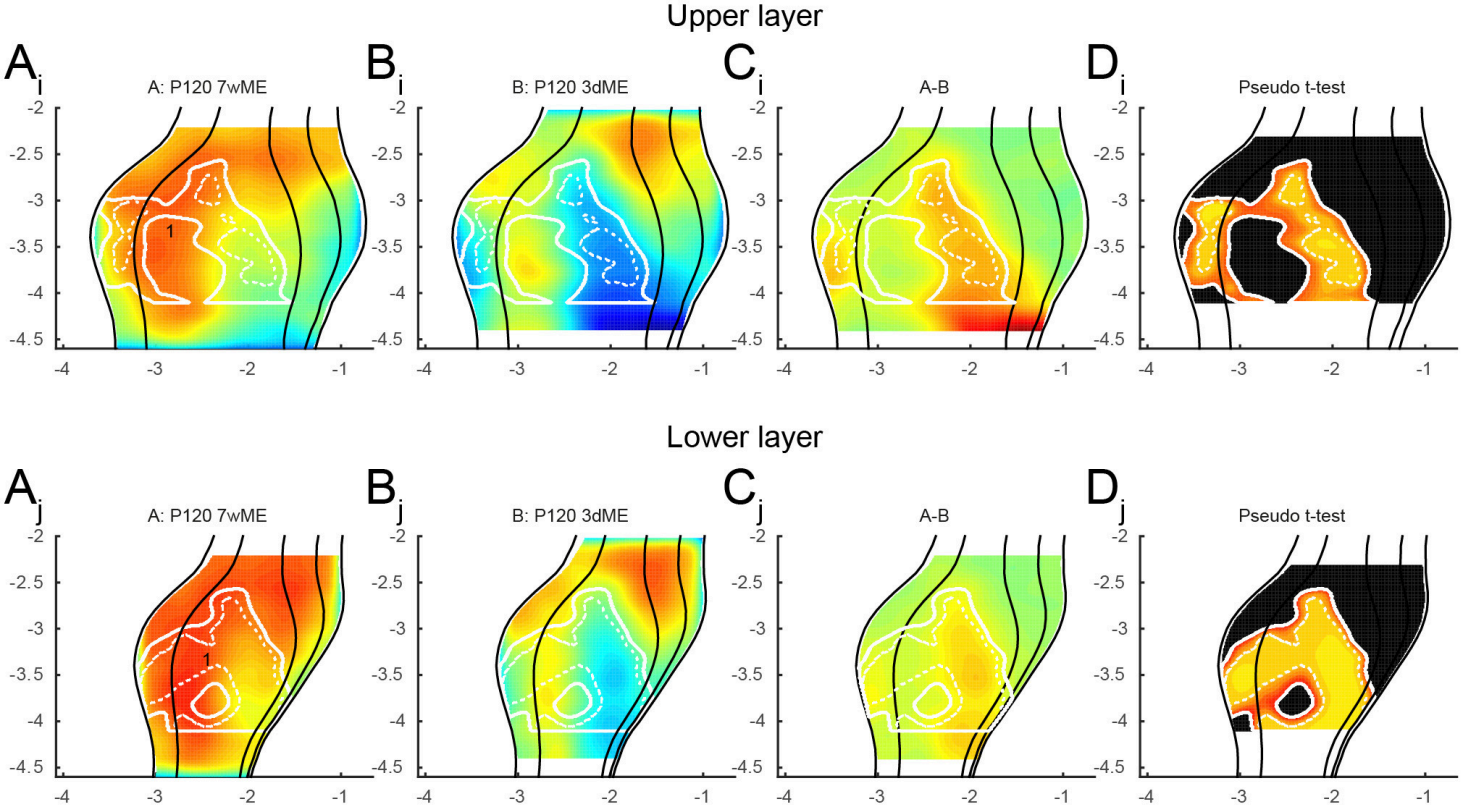
**Recovery of the visual cortex between 3 days and 7 weeks of enucleation for the (A_i_ − D_i_)** upper and **(A_j_ − D_j_)** lower layers. The upper layer shows no reactivation in the binocular zone of V1. The lower layer recovers quite similar to the upper layer, except for (1) the central binocular zone of V1. Panels and axes legends are the same as in Figure 4.

**Figure 7:**
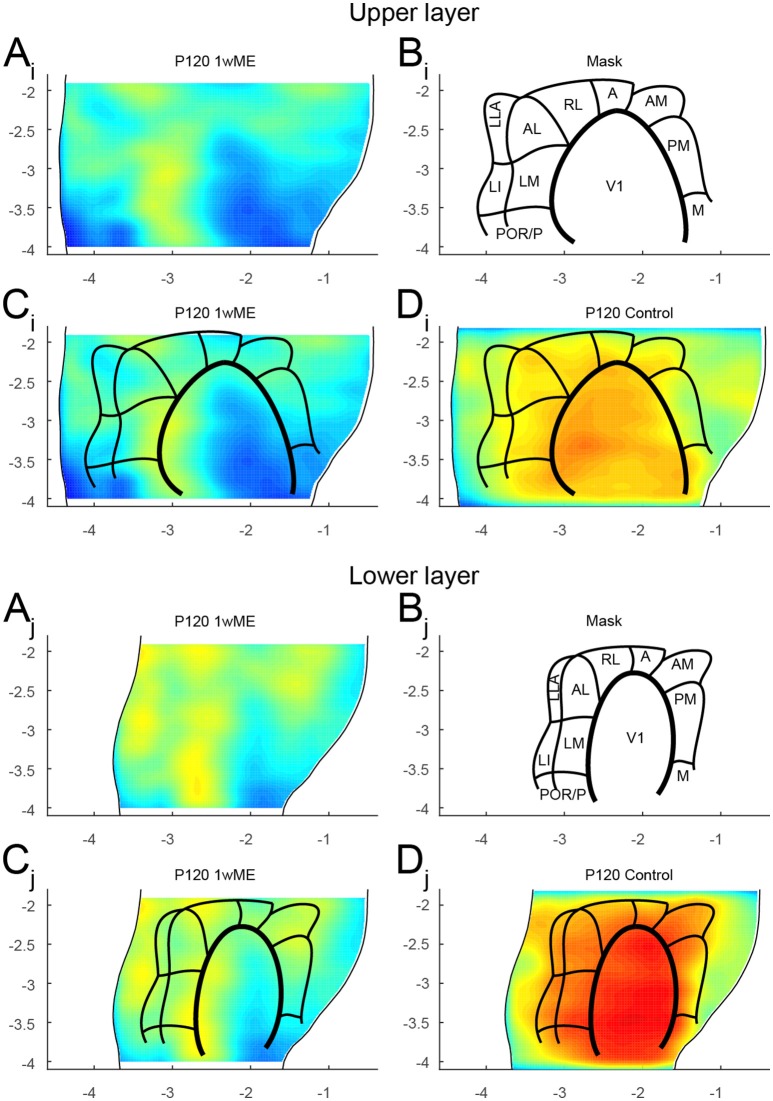
**Mapping of the extrastriate areas**. The extrastriate areas recently described in the mouse (Wang et al., 2007; Wang and Burkhalter, 2007; Garrett et al., 2014) could be delineated for the **(A_i_ − D_i_)** upper and **(A_j_ − D_j_)** lower layers using **(A)** the created top view of *zif268* expression patterns of 1wME mice which still heavily relies on the remaining ipsilateral eye inputs to drive the binocular zones. The outer lateral and medial borders were set at the rhinal fissure and the medial border of V2MM (red arrows in Figure 1A). **(B–C)** This creates a mask for all 11 extrastriate areas [lateromedial area (LM), anterolateral area (AL), rostrolateral area (RL), anterior area (A), anteromedial area (AM), posteromedial area (PM), posterior area (P), postrhinal area (POR), laterointermediate area (LI), laterolateral anterior area (LLA), and medial area (M)] and V1. **(D)** Overlaying the mask with sighted control animals results in a very accurate mapping of the visual areas with V1 having the highest *zif268* expression. Panels and axes legends are the same as in Figure 4.

We provide example data from *in situ* hybridization experiments for *zif268* on coronal brain slices of adult (P120) control mice or monocular enucleated (ME) animals with different survival times. Table 1 provides an overview of the specific parameters of the biological example data, which were applied to the generalized method to create Figures 1–**7**.

#### Animals

C57Bl/6J mice of either sex (*N* = 26) were obtained from Janvier Labs (Le Genest-St-Isle, France) and housed under standard laboratory conditions (i.e., standard cages with nesting material) under an 11/13-h dark/light cycle with food and water available *ad libitum*. All experiments were approved by the ethical research committee of the KU Leuven and were in accordance with the Declaration of Helsinki. Every possible effort was made to minimize animal suffering and to reduce the number of animals.

ME was performed in adult C57Bl/6J mice on postnatal day P120. We surgically removed the right eye as previously described in depth (Aerts et al., 2014a). Briefly, the animals were anesthetized with a mixture of ketamine hydrochloride (75 mg/kg; Dechra Veterinary Products; Eurovet) and medetomidine hydrochloride (1 mg/kg; Orion; Janssen Animal Health) in saline (i.p.) and the eyelids were disinfected with 70% ethanol. A sterile curved forceps was guided behind the eye to clamp the optic nerve. By making circular movements with the hand holding the forceps, the optic nerve was constricted in two. The animals were administered atipamezol hydrochloride (1 mg/kg; Orion; Elanco Animal Health) in saline (i.p.) to reverse the anesthesia, Meloxicam (1 mg/kg; Boehringer Ingelheim) as analgesic and eye ointment to prevent dehydration of the cornea.

We applied post enucleation survival times of 3 days and 1, 5, and 7 weeks (3dME and 1, 5, and 7wME; *N* = 4, 5, 5, 6 and Ctrl *N* = 6) after which we sacrificed the animals using an overdose of sodium pentobarbital (Nembutal; 600 mg/kg; Ceva Sante Animale) followed by cervical dislocation. The brains were quickly removed, flash frozen in 2-methylbutane (Merck) at a temperature of −40°C and stored at −80°C. 25 μm-thick coronal sections were cut on a cryostat (Microm HM 500 OM, Walldorf, Germany) and mounted on poly-L-lysine (0.1%) coated glass slides.

#### *In situ* hybridization for zif268

We performed *in situ* hybridization for *zif268* as described before (van Brussel et al., 2009; Van Brussel et al., 2011; Nys et al., 2014; Aerts et al., 2014b; Smolders et al., 2015). A series of coronal sections between Bregma levels −2.0 and −4.4 mm were selected to span the striate and extrastriate areas. We labeled the 3′-end of the mouse-specific synthetic oligonucleotide probe (5′-ccgttgctcagcagcatcatctcctccagyttrgggtagttgtcc-3′, Eurogentec, Seraing, Belgium) with [^33^P]dATP using terminal deoxynucleotidyl transferase (Invitrogen, Paisley, UK). Unincorporated nucleotides were separated from the labeled probe with mini-Quick SpinTM Oligo Columns (Roche Diagnostics, Brussels, Belgium). The radioactively labeled probe was added to a hybridization cocktail (50% (vol/vol) formamide, 4x standard saline sodium citrate buffer, 1x Denhardt's solution, 10% (wt/vol) dextran sulfate, 100 μg/mL herring sperm DNA, 250 μg/mL tRNA, 60 mM dithiothreitol, 1% (wt/vol) N-lauroyl sarcosine, 20 mM NaHPO_4_, pH 7.4) and applied to the cryostat sections (10^6^ c.p.m. per section) for an overnight incubation at 37°C in a humid chamber. The following day, sections were rinsed in 1x standard saline sodium citrate buffer at 42°C, dehydrated, air-dried and exposed to an autoradiographic film (Biomax MR, Kodak). Films for *zif268* were developed in Kodak D19 developing solution after 6 days. Fixation was performed in Rapid fixer (Ilford Hypam, Kodak). Autoradiographic images from the sections were scanned at 1200 dpi (CanoScan LIDE 600F, Canon).

#### Histological borders of visual areas

All sections were counterstained with 1% cresyl violet (Fluka Chemical; Sigma-Aldrich) according to standard protocols. Cresyl violet stainings provide sufficient information to delineate the primary visual cortex (V1), lateral extrastriate cortex (V2L), medio-lateral extrastriate cortex (V2ML) and medio-medial extrastriate cortex (V2MM) based on the cytoarchitecture as described in detail previously (Caviness, 1975; van Brussel et al., 2009; Van Brussel et al., 2011; Nys et al., 2014, 2015; Smolders et al., 2015) and comparisons were made with the stereotaxic mouse brain atlas (Paxinos and Franklin, 2013). The border annotations for each section were superimposed onto the corresponding autoradiographic image with Adobe Photoshop CS6 before being imported in Matlab (Figure 1A) to aid interpretation of areal borders in the created top views.

### Example data set 2: immunofluorescent staining

**Figure 8:**
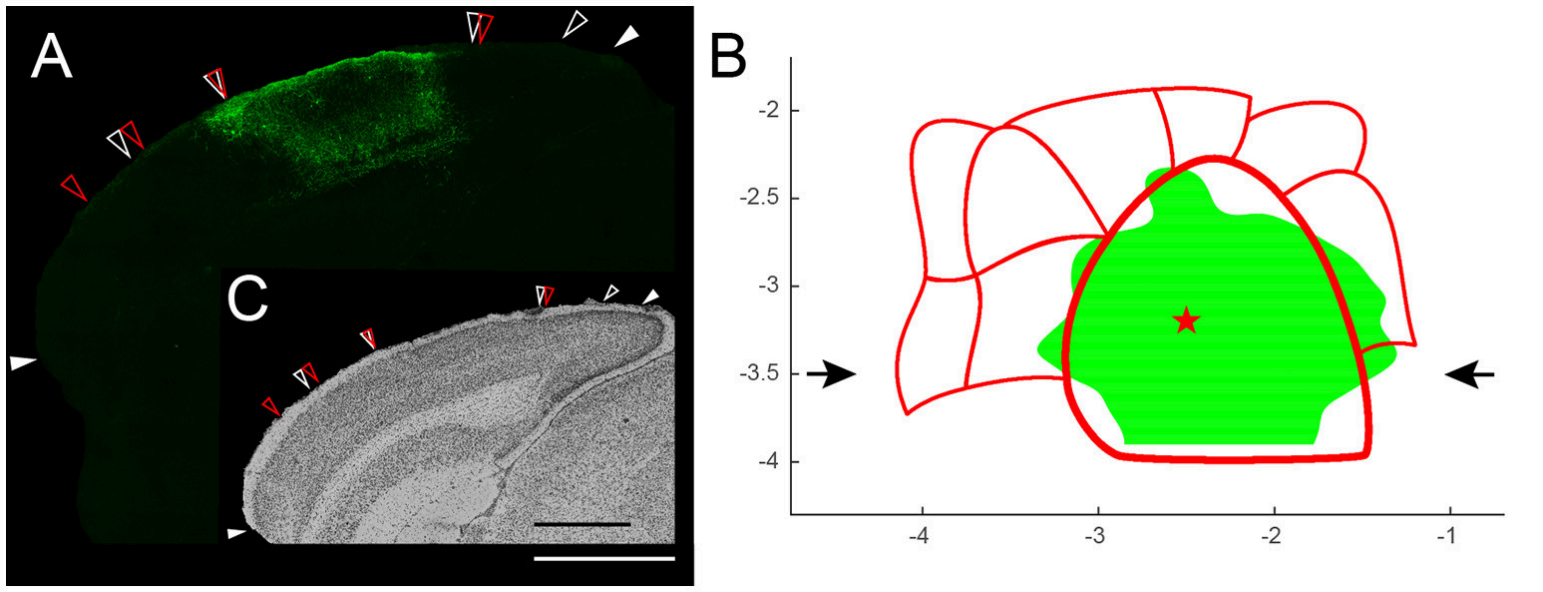
**(A)** Illustration of the expression pattern of SSFO-mCherry fusion protein in somatostatin (SOM)-positive cells in a coronal slice (bregma level −3.5 mm) after transducing SOM-Cre mice with rAAV2/7-CMV-FLEX-SSFO-mCherry in the primary visual cortex (center injection site: red star in B). Solid white arrows indicate the histological landmarks: the rhinal fissure and the medial border of V2MM (based on Nissl). White scale bar is 1 mm. **(B)** Top view of the spread of the viral vector transduction across the visual cortex thresholded by the upper quartile of the intensity distribution. Red star represents the center of the injection site (−3.2 mm posterior to bregma, −2.5 mm lateral to the midline, 400 μm from the pial surface). Black arrows mark the position of the slice shown in **(A,C)**. **(C)** Nissl staining: open white arrows indicate Nissl-based areal borders; open red arrows indicate the extrastriate areas' mask derived borders. Open arrows were transferred onto expression pattern image **(A)** confirming that most of V1 was transduced with only little spread to adjacent extrastriate cortices. Black scale bar is 1 mm.

To illustrate the applicability of this tool in interpreting different types of signals, we provide a second example dataset from immunohistochemically stained coronal brain slices of adult mice. Viral vector transduction in the primary visual cortex of these animals established the expression of a light-activatable opsin for optogenetic experiments. To assess whether the opsins are present only within the area of interest, immunohistochemical amplification of the fluorescent reporter protein fused to the opsin was analyzed using the top view representation tool. Table 1 provides an overview of the specific parameters of the biological example data, which were applied to the generalized method to create **Figure 8**.

#### Viral vector injections

The viral vectors were produced at the Leuven Viral Vector Core as previously described (Van der Perren et al., 2011). The recombinant adeno-associated viral vector (rAAV) 2/7 was used containing the inverted terminal repeats (ITRs) of rAAV2 and the capsid of rAAV7, as this serotype results in a proper expression pattern within the region of interest, i.e., the primary visual cortex (V1), without spreading into other nearby sensory areas (Scheyltjens et al., 2015). The rAAV2/7 viral vectors contain the cytomegalovirus promoter (CMV) to achieve strong expression in the mouse cortex and carry a transgene, in this case the stable-step function opsin [SSFO, C128S/D156A mutant of Channelrhodopsin-2 (ChR2)] between two pairs of incompatible lox-sites, resulting in a Cre-dependent transcription mechanism. The SSFO-gene is fused to a gene encoding the fluorescent protein mCherry, which will be built into the cell membrane together with the opsin for detection purposes. The genomic titer of the vector AAV2/7-CMV-FLEX-SSFO-mCherry corresponds to 8.55 × 10^11^ genome copies (GC) per milliliter.

Viral vector injections were performed in adult somatostatin (SOM)-Cre mice (STOCK Sst^tm2.1(Cre)Zjh^/J mice, The Jackson Laboratory; P90) anesthetized by intraperitoneal injections of a mixture of ketamine hydrochloride (75 mg/kg; Dechra Veterinary Products; Eurovet) and medetomidine hydrochloride (1 mg/kg; Orion; Janssen Animal Health). A small craniotomy above the primary visual cortex (V1) of the left hemisphere was created (−3.2 mm from bregma, 2.5 mm lateral to the midline, 400 μm from the pial surface) to inject (Nanoject II Auto-Nanoliter Injector, Drummond Scientific, Broomall, PA) a total volume of 600 nl of the viral vector (3 × 200 nl, 13.8 nl per steps, every 30 s) at 400 μm depth using a glass capillary (~20 μm tip diameter). Injections were performed around the center of the craniotomy, at approximately 170 μm triangularly distributed locations. After each injection, the capillary was left in place for an additional 2 min before being slowly retracted. Following wound suturing, the anesthesia was reversed by intraperitoneal injection of atipamezol hydrochloride (1 mg/kg; Orion; Elanco Animal Health).

At least 4 weeks after viral vector injections, when SSFO-mCherry-expression reaches maximal levels, the animals were sacrificed using an overdose of sodium pentobarbital (Nembutal; 600 mg/kg; Ceva Sante Animale) followed by cervical dislocation. The brains were quickly removed, flash frozen in 2-methylbutane (Merck) at a temperature of −40°C and stored at −80°C. 25 μm-thick coronal sections were cut on a cryostat (Microm HM 500 OM, Walldorf, Germany) and mounted on poly-L-lysine (0.1%) coated glass slides.

#### Immunohistochemistry

Immunofluorescent stainings for mCherry were performed on a series of post-fixed (4% PFA in PBS) cryosections. After washing the sections in PBS and incubation with normal goat serum (Chemicon) for 45 min, the sections were incubated over a period of 24 h with the primary antibody, polyclonal rabbit anti-red fluorescent protein (RFP) (Rockland Immunochemicals, ab600-401-379), diluted 1:5000 in Tris-NaCl blocking buffer (TNB). After rinsing in PBS the sections were incubated with the secondary antibody, Alexa Fluor 594 (polyclonal goat anti-rabbit IgG, Life Technologies, A11011, Ghent, Belgium) diluted 1:250 in TNB for 2 h and were then counterstained with DAPI (2 μL/100 mL PBS, Sigma-Aldrich, 3260) before coverslipping with Mowiol solution. Overview images of each cortical slice containing the immunofluorescent signal were made with an inverted FV1000 confocal microscope (IX81, Olympus, Aartselaar, Belgium) using a 20x objective (NA 0.75) at a resolution of 512 × 512. Pictures were taken as z-stacks at 3-μm intervals covering the entire thickness of the section (25 μm).

## Results

The newly developed method was first optimized and validated using the data from an *in situ* hybridization-based analysis of expression changes for the activity reporter gene *zif268* in the visual cortex of adult mice of different post mono-enucleation survival times. Mice were analyzed between 3 days post enucleation, when no recovery has yet taken place and the lesion therefore presents its maximal impact, up to 7 weeks, a time point when maximal recovery of neuronal activity has been reported (Van Brussel et al., 2011; Nys et al., 2014). In addition to confirming what was already known in terms of recovery, the new method described here allowed for the detection and quantification of changes throughout the entire visual cortex. Indeed, all slices from −2.0 to −4.4 mm to bregma, with a fixed inter-slice interval of 0.1 mm, were manually selected by cross-referencing with the mouse brain atlas (Paxinos and Franklin, 2013) and included in the present study, allowing the analysis of V1 as well as extrastriate cortical territory lateral, medial and anterior of V1 in one rendering. The contralateral visual cortex between the lateral border of the lateral extrastriate cortex (V2L) and the medial border of the medio-medial extrastriate cortex (V2MM) was examined using *K* = 30 segments spanning the full depth of the six cortical layers (Figures 1, 2) creating top view representations of the *zif268* gene expression for each of the experimental conditions (Figure 3), with interareal borders superimposed based on histology (Nissl) as histological landmarks. The calculated non-uniform sampling distribution following the projection to the horizontal plane was 0.100 mm for the upper quartile and 0.143 for the upper fence for medio-lateral resolution, related to the variation in brain width (Table 1).

### Qualitative observations

The *zif268* expression level in sighted control mice (Figures 3A,B) reflects maximal and uniform neuronal activity within the primary visual cortex and within V2L. In the medio-lateral extrastriate cortex (V2ML) and V2MM a gradient of expression is observed both along the antero-posterior and the medio-lateral axis, with a maximal expression in its postero-lateral subdivision, and lower expression levels confined to its anterior half.

In enucleated mice, *zif268* expression patterns are drastically affected in comparison to sighted control mice (Figures 3A–F). The most pronounced effect of enucleation is the clear decrease in signal intensity observed in the medial half of V1, an area corresponding to the V1 monocular zone that normally processes inputs from the lost eye (see illustration Figure 3B). In the extrastriate areas, surrounding V1, several additional small patches of low *zif268* expression are found which may represent the monocular zones within the extrastriate areas. At 3 days post-enucleation (Figure 3C), expression levels of *zif268* are very low in these putative monocular zones. At 7 weeks post-ME (Figure 3F), the levels of *zif268* indeed appear higher again, yet they do not seem to fully recover up to normal values, at least not throughout the full extent of the visual cortex. Especially the posteromedial subregion of visual cortex still displays lower activity levels compared to normal controls (Figures 3A,F).

### Quantitative analysis

To precisely chart if cortical subregion differences within V1, V2L, V2ML, and V2MM exist due to the loss of contralateral visual inputs, the top view images of enucleated mice were compared with the ones of sighted control mice using a pseudo *t* -test. Figure 4 respectively gives an overview of the experimental conditions (condition A; Figures 4A_i-iv_) and control animals (condition B; Figures 4B_i-iv_), illustrates the difference between these conditions A and B (Figures 4C_i-iv_) and the statistical relevance of this difference using a pseudo *t* -test (Figures 4D_i-iv_). The contour of the result of the pseudo *t* -test is overlaid in all panels for the ease of interpretation. This analysis revealed the most profound effect of monocular enucleation in the posterior aspect of the visual cortex, where *zif268* levels are significantly reduced in all four cortical regions (Figure 4D_i_—red to yellow gradient area). The posterior part of all four areas recovers over time except for the monocular zone of V1 and extrastriate cortex medial to V1, which remains hypoactive even 7 weeks after enucleation. In all conditions, the posterior monocular zone accounts for most of the overall decrease in *zif268* expression levels.

In the anterior aspect of the visual cortex, no significant effect of the visual deprivation is observed, except at 1-week post-enucleation, where a delayed suppression, that swiftly recovers, is found (Figure 4D_ii_).

In V2L, a patch of decreased *zif268* expression initially spreads over more than 1 mm in the antero-posterior axis, but it gradually reduces in size until it almost disappears at 7wME (Figures 4A_iv_–D_iv_).

In V2ML and V2MM, the posterior half of the area remains affected by the enucleation. Although this area gradually recovers over time, *zif268* levels in this part of the visual cortex never reach normal values again at time points investigated in this study.

In all visual areas, expression levels in the binocular zones are less reduced, most likely because of the remaining inputs from the ipsilateral eye (Figures 4A_i-iv_,D_i-iv_). There is an effect at 3dME in the binocular zone of V1 and V2L, but it quickly recovers within 1 week and becomes completely reactivated after 5 weeks. This recovery in the binocular zone co-occurs with a medial expansion toward the monocular zone of V1.

It has been reported that the reactivation of the visual cortex after monocular enucleation comes in two waves and is different for supragranular and infragranular layers (Van Brussel et al., 2011). To further characterize these waves, the data was split to analyze differences in recovery separately in the upper (layers I to IV; Figures 5A_i, ii_) and lower (layers V-VI; Figures 5A_j, jj_) layers. In general, *zif268* expression levels are quite similar between upper and lower layers and closely resemble what has been previously described. The layer-based subdivisions have a similar activity pattern at 3dME with a marked decrease in expression throughout the visual cortex, except for the most anterior cortical regions. The gradual recovery until 7wME, leading to the medial expansion of the binocular zone into the monocular segment of V1, is also present in upper and lower layers (Figure 5A). There are three subregions that display a layer-effect of enucleation. First, in the upper layers, there is a patch in the posterior part of the binocular zone of V1 (Figure 5A_i_; labeled “1”) that is not affected by enucleation at 3 days. Second, there is a patch of reduced *zif268* expression in V2L (Figure 5A_ii_; labeled “2”) that does not recover over time in the upper layers. Third, although it is initially found at a similar antero-posterior position in both layers, there is a patch in the medial extrastriate areas that is found more posterior in the lower layers than in the upper layers in 7wME mice (Figures 5A_i, ii, j, jj_; labeled “3”).

To further investigate the recovery of the visual cortex over time, the upper and lower layer maps were compared between the 3dME and 7wME mice (Figure 6). In the upper layers, the reactivation occurs over the whole antero-posterior extent of the monocular zone in V1 and in the posterior half of V2L. In the binocular zone of V1, no significant difference is found between animals enucleated for 3 days and 7 weeks. In the lower layers, the recovery is quite similar to the one in the upper layers except for the central binocular zone of V1 (Figure 6; labeled “1”) where higher *zif268* levels were found at 7 weeks ME.

### Mapping extrastriate areas

To validate the power of the spatial resolution of this newly developed mapping tool, we next tested if the extrastriate areas recently described in the mouse based on tracer and optical imaging data (Wang et al., 2007; Wang and Burkhalter, 2007; Garrett et al., 2014) could be delineated using the created top view of *zif268* expression patterns. Because the recovery of activity is incomplete after 1wME and the recorded activity still heavily relies on the remaining ipsilateral eye inputs only (Van Brussel et al., 2011), the activity patches identified by high *zif268* levels should only be centered above binocular cortical zones. Based on the assumption that almost all extrastriate areas have a complete visual field representation, including both the monocular and binocular visual fields, a detailed map of the extrastriate areas was drawn based on the distribution of the binocular zones for upper and lower layers separately (Figure 7). In this case, only two borders were traced in the “slice registration” step: one at the rhinal fissure and one at the medial border of area V2MM (red arrows in Figure 1A). This was done for 1wME mice and also for sighted control mice. The slices were divided in 50 segments resulting in a resolution distribution along the medio-lateral axis of 0.096 mm (upper quartile) and 0.187 mm (upper fence) (Table 1).

The top view activity maps of 1wME mice clearly show such regions with higher *zif268* expression levels, indicating the location of the different binocular zones in the map (Figure 7A, yellow). Using this approach, and by cross-referencing to previously published maps of Wang and Burkhalter (2007) and of Garrett et al. (2014), it was possible to delineate the borders of V1 and of 11 extrastriate areas: lateromedial area (LM), anterolateral area (AL), rostrolateral area (RL), anterior area (A), anteromedial area (AM), posteromedial area (PM), posterior area (P), postrhinal area (POR), laterointermediate area (LI), laterolateral anterior area (LLA), and medial area (M) to generate an extrastriate-border mask for the upper and lower layers separately (Figures 7B,C). The extrastriate-border mask drawn on the maps from the 1wME mice (Figure 7C) was then transferred onto the map from the sighted control mice (Figure 7D). This resulted in a very accurate mapping of the visual areas in control mice, with V1 having the highest *zif268* expression.

### Viral vector transduction pattern

To illustrate the broader applicability of this projection tool, we provide a second example, now from immunohistochemically stained coronal brain slices. In adult SOM-Cre mice injected with an rAAV2/7-CMV-FLEX-SSFO-mCherry viral vector, only the SOM-positive cells were transduced to express the opsin SSFO fused to the fluorescent reporter mCherry. The latter tag was immunohistochemically detected and amplified (Figure 8A).

The goal was to apply a triple injection approach to transduce most of V1 without affecting extrastriate areas. All slices spanning the visual cortex were combined to create the top view image, which was then thresholded for the 25% highest intensity values. After placing the previously generated coordinate-based mask for the delineation of the borders of all visual areas without any rescaling or translation, it became evident that the injections resulted in a broad transduction pattern across the entire primary visual cortex with only little spread to adjacent extrastriate cortices (Figure 8B). The top view representation therefore allowed us to confirm that our approach was successful. Furthermore, this interpretation has been double checked by regular histology analysis of the transduction site (Nissl; Figure 8A inset).

## Discussion

### Technical considerations and limitations

The newly developed method representing OD values qualitatively or quantitatively from series of sections in a plane of interest, often the horizontal plane for brain, performs very well on mouse brain because of its non-gyrated structure and lack of strong curvature. In the case of highly gyrated brains however, the presence of the many circumvolutions with areas where the brain tissue folds onto itself will make it difficult to project the data onto a single plane of view. In this specific example, two or more measurements would be performed at the exact same position on the medio-lateral axis. When projected to the plane of view, all measurement from that medio-lateral position would be merged together on the corresponding medio-lateral position of that plane, resulting in a significant loss of information and in possible misinterpretations. Although it is possible to follow the curvature of the gyri and sulci for a detailed analysis, we certainly do not recommend doing so when the end result is a top view representation. Instead, we would advise to use a “jumping across” approach, where the gyri are excluded during the delineation step of the top and bottom edges. The resulting figure would then show the gyri as a drop in signal, which would closely resemble to the output of optical, flavoprotein and Ca^2+^ imaging methods when imaging the same region *in vivo*.

Another disadvantage of integrating highly gyrated structures or structures with a strong curvature in at least one end is that there will be a less accurate representation of that end in the plane of view because of the smoothing algorithm, which is based on the second derivative. To avoid clipping the borders of the areas of interest (at both ends of the top and bottom edges) during the calculation of the second derivative, the last points of both ends are mirrored. In the case of strongly curved structures, mirroring the curvature would result in a bump that would be recognized as a deviation of the overall curve. This point would therefore be flattened out, resulting in a broadened zone compared to its actual dimensions.

The smoothing algorithm based on the second derivative which is applied at different steps during the creation of the reference map penalizes points that deviate from their neighboring points (blue markers in Figure 2C). Using this algorithm allows us to compensate to some extend for different kinds of subject- and user-based errors that can occur, e.g., distorted morphology of the slice, errors in assigning histological landmarks or the reference line or small differences in cutting angle between animals, to create a rigorous reference map with histological landmarks.

Finally, the proposed method also allows for statistical comparisons of the maps between two experimental groups but this requires high computational power. Indeed, a pseudo *t* -test is performed by calculating the *t*-statistic for all possible permutations between the animals within two conditions. In order to detect significant differences, each experimental group must have at least three animals per group to perform the minimum number of permutations to reach a *p* -value of 0.05. It is possible to increase the statistical power and to detect *p* -values of 0.01 or even 0.001, but this will require increasing the number of animals. A total of six animals per group seems however to be the limit as adding more animals exponentially increases the computational burden (Table 2).

### Biological significance

With the new method presented here, we were capable of creating top view mouse brain images consistent and complementary with optical imaging, flavoprotein imaging or Ca^2+^ imaging. Optical density measurements of the expression of *zif268*, a neuronal activity marker (Arckens et al., 2000a,b; Hu et al., 2009; van Brussel et al., 2009; Van Brussel et al., 2011; Nys et al., 2014; Smolders et al., 2015), allowed the generation of visual activity maps for sighted and ME mice after different post mono-enucleation survival times (Figure 4). Over the time course of 7 weeks, the posterior regions of the striate and extrastriate areas recovered readily, except for the posterior monocular zone of V1 and medial extrastriate cortex. This observed plasticity phenomenon is consistent with earlier publications (Van Brussel et al., 2011; Nys et al., 2014). With this new approach, three patches with a deviating recovery pattern were identified that had not been described before (Figure 5).

The first two patches were found in the upper layer at the V1/V2L border, in the binocular zone (Figures 5A_i, ii_; labeled “1” and “2”). The first patch was found in the posterior part of the binocular zone, whereas the second one was found more anteriorly. The first one (posterior) was not affected by 3 days of ME, but the second one (anterior) was and it did not recover over time. Because this occurred within the binocular zone of V1, it is possible that the remaining ipsilateral eye provided sufficient input to limit the drop in activity in the posterior patch, but it does not explain the lack of recovery in the most anterior patch. Another explanation could be that these patches fall into different eye-specific domains that have been described in the binocular zone of rats (Laing et al., 2015). The first patch, which is not affected by ME, could be situated in a domain corresponding to the ipsilateral eye, which would continue to receive visual inputs even after monocular enucleation of the contralateral eye. The second patch, on the other hand, could be in a domain linked to the contralateral eye and would thus be highly affected by the monocular enucleation. Whether these eye-specific domains really exist in mice remains elusive, but it is very likely considering that the callosal pattern is quite consistent between mice and rats (Rhoades et al., 1984; Olavarria and Van Sluyters, 1995) and because these domains are determined by the distribution of callosal and acallosal patches (Laing et al., 2015). Because of the size of the mouse brain, one could expect that these eye-specific domains are quite small. The fact that relatively big patches were observed here might be due to the limitations of the method in terms of spatial resolution, merging smaller patches together.

The last patch was found in the medial extrastriate areas and in a more posterior position in the lower layer than in the upper layer at 7 weeks post-enucleation (Figures 5A_i, ii, j, jj_; labeled “3”). Previous observations have shown that the recovery of the monocular visual cortex is mainly driven by somatosensory inputs and this mainly in the lower layer (Van Brussel et al., 2011). Because the somatosensory cortex is located anteriorly to the visual areas, it is not surprising to find a more important recovery expanding antero-posteriorly in the lower layer than in the upper layer.

### A map of extrastriate areas

In 1wME mice, inputs from the remaining ipsilateral eye result in islands of higher *zif268* expression, corresponding to the location of the binocular zones. Based on the assumption that the visual field representation is almost complete in all extrastriate areas, it was therefore possible to delineate the borders of the 11 extrastriate areas previously described, based on anatomical evidences (Coogan and Burkhalter, 1993; Wang et al., 2007; Wang and Burkhalter, 2007) and on optical imaging (Schuett et al., 2002; Garrett et al., 2014) (Figure 8B). Generally, the borders that were traced using the activity maps from 1wME mice correspond well to the borders of V1, V2L, V2ML, and V2MM traced based on the cytoarchitecture (Nissl staining). Furthermore, the extrastriate map drawn based on the activity in 1wME mice reconciles the most recent extrastriate maps (Wang and Burkhalter, 2007; Garrett et al., 2014). Indeed, in the map of Garrett et al. (2014), area A was not consistently observed, whereas that area was very well described in the map of Wang and Burkhalter (2007). On the other hand, areas LLA and M were also identified here, which is in agreement with the map of Garrett et al. (2014). Finally, by comparing our map with the schematic illustration proposed by Van der Gucht et al. (2007), it appears that area AM corresponds with areas RM1 and RM2 and that areas AL, RL and A correspond with the area V2AL. This would indicate that areas RM3 and RM4 are not linked to any extrastriate area and this would fit very well with the idea that RM1 and RM2 are vision driven areas, whereas RM3 and RM4 are more multimodal driven (Van der Gucht et al., 2007).

### Permutation test with pseudo *t*-statistics

A permutation test was used to compare the conditions one by one. Based on the assumption that the null hypothesis is true, being that no difference can be found between the different conditions, one could randomly sort the maps from each mouse in either group and the end result would be the absence of differences between the conditions. In the case where a significant difference could be found in one or more sets of permutations, this would indicate that the animals are not similar and that there are differences between the groups. The advantage of using a permutation test for the comparison of images is the fact that complete 2D images can be compared and not just regions of interest (Fisher, 1935; Pitman, 1937; Edgington, 1964; Holmes et al., 1996; Sprent and Smeeton, 2001). However, because the number of permutations depends on the total number of animals present in each experimental group, and because a minimum of 20 permutations are needed to detect at least one significant pixel (Holmes et al., 1996), the minimum number of animals that can be used is three per condition. In the dataset presented here, comparisons were made on groups ranging from four to six mice, which resulted in a sufficient number of permutations to successfully detect areas containing significantly different pixels between conditions with a *p* -value smaller than 0.01, as represented by the dashed line in Figures 4–6.

A pseudo *t* -test was implemented as statistical image in this permutation test. The advantage of using a pseudo *t* -test over a more common *t* -test lies in the smoothing of the variance in the population to reduce detection of false positives. This is particularly important when using small group sizes (Fisher, 1935; Holmes et al., 1996). It is important to note that all the animals in the dataset must have a similar coverage in the antero-posterior and medio-lateral axes. Failing to do so, will result in a clipped statistical image and *p* -value contour lines (see Figure 5C), because we tend to keep the amount of data points (N and M) constant to stabilize the variation. The maximal size of the map is therefore limited to the smallest map in the dataset.

### A versatile technique

In this paper, coronal sections processed for *in situ* hybridization were used to apply, optimize and validate this new method of data presentation and analysis. Although our choice was made toward this specific dataset because of our molecular enucleation model and of our previous results (Van Brussel et al., 2011; Nys et al., 2014, 2015), the method is certainly not limited to brain tissue, coronal sections or to *in situ* hybridization data. First, any tissue or organ (kidney, heart, brain…) of any species (animal or plant) is suitable within the limitations of the applied imaging technique. Having an anatomical or molecular reference atlas for this specific tissue can be helpful to determine the histological landmarks which have to be manually assigned by the user during the slice registration step to define the outer boundaries of the region of interest. However, if no such atlas exists, any clearly visible histological landmark, i.e., a gyrus, that stretches the region of interest can be used to define these outer boundaries. Second, not only coronal sections, but also sagittal, horizontal or even tangential sections could be used as input as any projection plane can be calculated by simply adapting the cutting plane, choice of reference line and the mathematics. Third, any other optical density-based dataset from immunohistochemistry, fluorescent or bright field images, as illustrated with the mapping of the second exogenous fluorescence dataset (Figure 8), could also be used as input as long as they are adapted to gray scale images prior to the analysis. Finally, several markers and techniques could be applied to the same animal on adjacent sections to increase the amount of information collected per animal. In this paper, four series of 25 μm serial sections were prepared in relation to the *zif268* ISH analysis. This means that three other specific RNA or protein markers could easily be added to this dataset by using the remaining series. Furthermore, decreasing the cutting thickness and optimizing the staining techniques for double or even triple staining could potentially increase the number of parallel information even further. This opens the opportunity to combine several techniques to better link functional, anatomical and molecular information.

## Conclusion

The newly developed mapping method is powerful and versatile in projecting series of images to a planar representation either qualitatively or quantitatively via statistical comparison of such images of different conditions using a pseudo *t* -test. We applied the technique to an *in situ* hybridization dataset and to an immunocytochemistry dataset. We were able to confirm and extend previous research findings (Van Brussel et al., 2011; Nys et al., 2014). The representation technique further allowed the creation of a generic mask for all 11 extrastriate areas surrounding V1, applicable for future molecular mapping experiments. Together these examples are illustrative of the general applicability of the new tool in presenting and analyzing molecular datasets from different tissues from different species, independent of age at the time of analysis.

## Author contributions

All authors contributed to revising the work, had final approval of the version to be published and agree to be accountable in relation to the accuracy and integrity of the work. SV drafted the paper and SV, ML, and LA made substantial contributions to the conception and design of the work; SV, ML, and LA made substantial contributions to the interpretation of data and SV, ML, and IS made substantial contributions to the acquisition of data.

## Funding

This work was supported by grants of the Research Council KU Leuven and the Research Foundation—Flanders (FWO) (G061216N, G065913N). ML is a postdoctoral fellow of the FWO (12I7316N). IS is a Ph.D. fellow of Agency for Innovation by Science and Technology (IWT, 121240).

### Conflict of interest statement

The authors declare that the research was conducted in the absence of any commercial or financial relationships that could be construed as a potential conflict of interest.

## Supplementary material

Supplementary Figure 1 **Validation randomization test with pseudo ***t*** statistics. (A)** The control condition (*N* = 6) was split in 2 groups A and B (*N* = 3) and compared. Pseudo *t* statistics detected no *p* -values below α/2 = 0.025, confirming the hypothesis that there are no significant differences within a certain condition. Column 1 and 2 display the split conditions A and B, column 3 represents the relative difference between condition A and B and column 4 and 5 show the adjusted *p* -value images for activation and deactivation respectively for the two tailed pseudo *t* -test, thresholded at *p* = α/2 = 0.025. **(B)** Normal distributed random artificial data was 1000 times generated (mean OD value = 50%, standard deviation = 20%, grid = 30 × 25) for each subject (*N* = 12). The subjects were split in two groups (*N* = 6) and compared within each repetition. One example of conditions A and B out of the 1000 repetitions are shown in column 1 and 2. Column 3 represents the relative difference between condition A and B. Column 4 and 5 show the distribution of the minimal *p* -value within the *p* -value image for activation and deactivation, respectively, for each of the 1000 repetitions. The family-wise error rate (FWER) was calculated as the percentage of these 1000 repetitions in which we could detect at least one significant pixel with *p*_*activation*_ ≤ α/2 = 0.025 and *p*_*deactivation*_ ≤ α/2 = 0.025. The FWER was (20 + 28)/1000 = 0.048 which matches closely the chosen alpha rate of 0.05. A pie chart illustrates the proportion false positive signals accounting to the FWER. This finding closely matches the observation described by Nichols and Holmes (2003) that this procedure maintains a strong control over the FWER.
