# Supplementary figures and images for: A Tool for Brain-Wide Quantitative Analysis of Molecular Data upon Projection into a Planar View of Choice
Source: Front Neuroanat. 2017 Jan 17;11:1. doi: 10.3389/fnana.2017.00001 (PMC5239821; doi:10.3389/fnana.2017.00001)

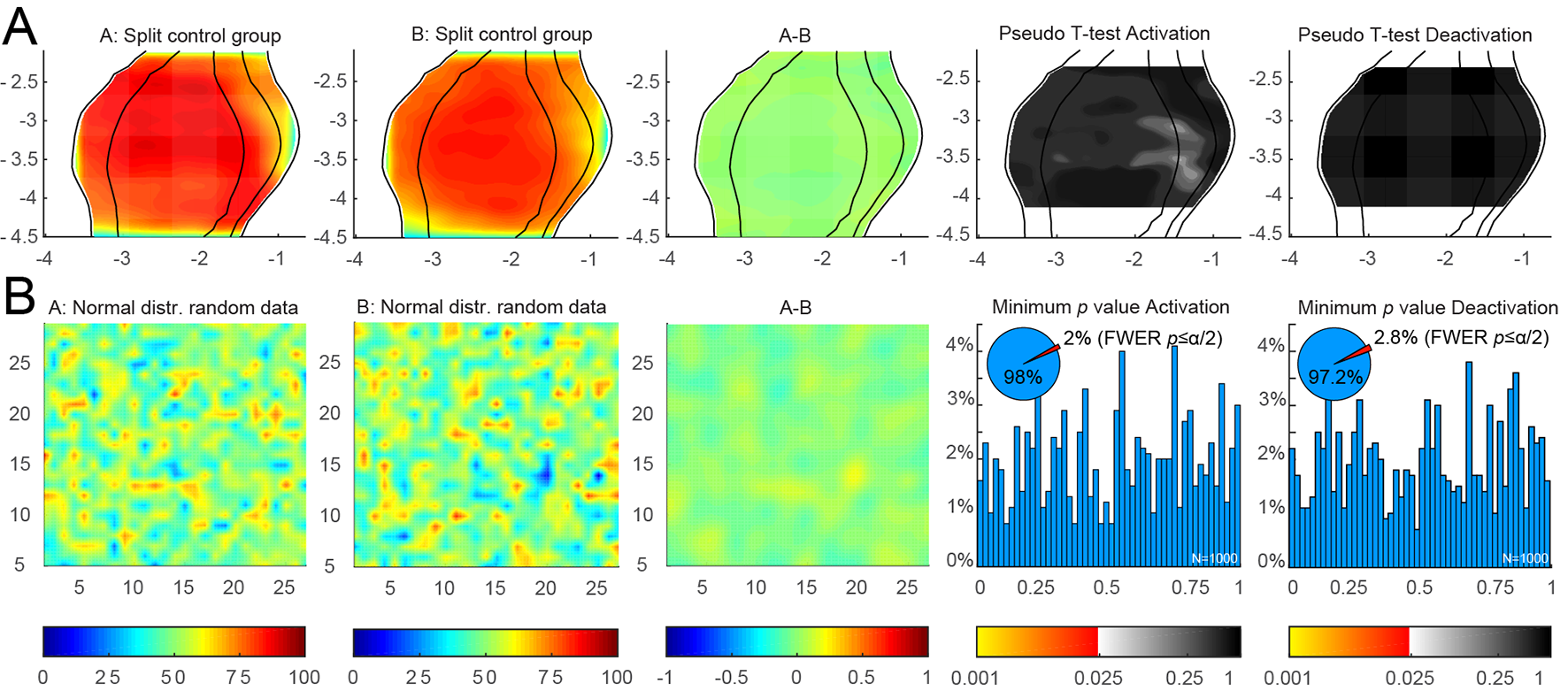

Supplement: Supplementary Figure 1 — Validation randomization test with pseudo t statistics. (A) The control condition (N = 6) was split in 2 groups A and B (N = 3) and compared. Pseudo t statistics detected no p -values below α/2 = 0.025, confirming the hypothesis that there are no significant differences within a certain condition. Column 1 and 2 display the split conditions A and B, column 3 represents the relative difference between condition A and B and column 4 and 5 show the adjusted p -value images for activation and deactivation respectively for the two tailed pseudo t -test, thresholded at p = α/2 = 0.025. (B) Normal distributed random artificial data was 1000 times generated (mean OD value = 50%, standard deviation = 20%, grid = 30 × 25) for each subject (N = 12). The subjects were split in two groups (N = 6) and compared within each repetition. One example of conditions A and B out of the 1000 repetitions are shown in column 1 and 2. Column 3 represents the relative difference between condition A and B. Column 4 and 5 show the distribution of the minimal p -value within the p -value image for activation and deactivation, respectively, for each of the 1000 repetitions. The family-wise error rate (FWER) was calculated as the percentage of these 1000 repetitions in which we could detect at least one significant pixel with pactivation ≤ α/2 = 0.025 and pdeactivation ≤ α/2 = 0.025. The FWER was (20 + 28)/1000 = 0.048 which matches closely the chosen alpha rate of 0.05. A pie chart illustrates the proportion false positive signals accounting to the FWER. This finding closely matches the observation described by Nichols and Holmes (2003) that this procedure maintains a strong control over the FWER. [file Image1.TIF]
